# EVALUATION OF A TECHNOLOGY-BASED EXERCISE PROGRAM FOR OLDER ADULTS IN THOSE WITH CHRONIC MEDICAL CONDITIONS

**DOI:** 10.1093/geroni/igad104.2813

**Published:** 2023-12-21

**Authors:** Sarah Lieber, Jerad Moxley, Joseph Sharit, Peter Pirolli, M Cary Reid, Sara Czaja

**Affiliations:** Hospital for Special Surgery, New York City, New York, United States; Weill Cornell Medicine, New York City, New York, United States; University of Miami, Miami, Florida, United States; Florida Institute for Human & Machine Cognition, Pensacola, Florida, United States; Weill Cornell Medicine, New York City, New York, United States; Weill Cornell Medicine, New York City, New York, United States

## Abstract

Although physical exercise is known to be beneficial, most older adults do not engage in recommended levels of physical activity, particularly those with chronic medical conditions. In an exploratory analysis, we evaluated the efficacy of a technology-based exercise program (Fittle) for sedentary community-dwelling adults ≥60 years of age with social support features in a subset of participants with ≥2 self-report chronic medical conditions. Participants in the Fittle condition received group training and were placed into teams to complete exercises at home via a computer tablet. They also were able to “chat” with other team members. Participants in the control condition were directed to exercise websites designed for older adults. A total of 181 participants with a mean age 70.1 years were randomized to the intervention or control condition. Participants were predominantly women (80.1%) and came from diverse backgrounds, with 13.4% self-identifying as Black or African American and 12.7% as Hispanic or Latino. Among 93 participants with ≥2 self-report chronic conditions, individuals in the intervention (versus control) arm described greater social support (t(162)=1.97, p=0.03) and less loneliness (t(163)=-1.97, p=0.03) at 6 months; there was a non-significant trend for higher self-report physical activity in the Fittle arm (t(166)=1.17, p=0.12). Fittle improved perceptions of social support and loneliness in older adults with chronic medical conditions. Further study is needed in a larger dedicated cohort of older adults with chronic medical conditions to determine Fittle’s efficacy in this population.

